# 1,2-Bis(pyridin-4-yl)diazene–3,4,5-trihy­droxy­benzoic acid–methanol (3/2/2)

**DOI:** 10.1107/S1600536812031029

**Published:** 2012-07-14

**Authors:** Elena Rusu, Sergiu Shova, Gheorghe Rusu

**Affiliations:** a Institute of Macromolecular Chemistry ‘Petru Poni’, Polymer Chemistry and Physics Department, 41A Grigore Ghica Voda Alley, Iasi-700487, Romania; b Institute of Applied Physics of the Academy of Science of Moldova, 5 Academiei Street, Chisinau MD-2028, Moldova; c Institute of Macromolecular Chemistry ‘Petru Poni’, Inorganic Polymers Department, 41A Grigore Ghica Voda Alley, Iasi-700487, Romania

## Abstract

The title compound, 3C_10_H_8_N_4_·2C_7_H_6_O_5_·2CH_4_O, has a mol­ecular crystal structure which results from the cocrystallization of gallic acid (GA), 4,4′-azodipyridine (AzPy) and methanol in a 2:3:2 molar ratio. The asymmetric unit comprises one molecule each of GA, AzPy and methanol in general positions and half a molecule of AzPy as this is located about a centre of inversion. In the crystal, all the components of the structure are associated *via* the extended system of hydrogen bonds (O—H⋯O and O—H⋯N) and π–π stacking inter­actions [centroid–centroid distance = 3.637 (3) Å] into two-dimensional supra­molecular layers which are packed parallel to the [101] plane. The shortest perpendicular distance and the slippage between aromatic groups are 3.395 (3) and 2.152 (3) Å, respectively. The AzPy mol­ecules display a *trans* conformation with respect to the azo groups.

## Related literature

For the photosensitive properties of azo compounds, see: Qiu *et al.* (2011 ▶). For potential applications of gallic acid, see: Fazary *et al.* (2009 ▶). For the synthesis and cocrystallization ability of 4,4′-azodipyridine, see: Launay *et al.* (1991 ▶); Zhuang *et al.* (2006 ▶); Kanoo *et al.* (2012 ▶).
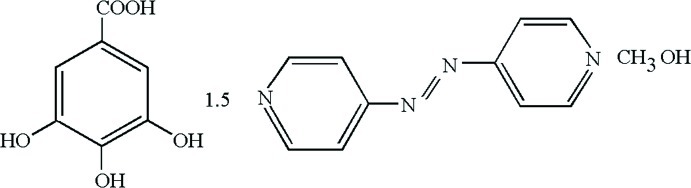


## Experimental

### 

#### Crystal data

3C_10_H_8_N_4_·2C_7_H_6_O_5_·2CH_4_O
*M* *_r_* = 956.93
Monoclinic, 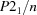

*a* = 13.555 (5) Å
*b* = 11.711 (5) Å
*c* = 14.213 (5) Å
β = 93.427 (5)°
*V* = 2252.2 (15) Å^3^
*Z* = 2
Mo *K*α radiation
μ = 0.11 mm^−1^
*T* = 200 K
0.2 × 0.2 × 0.1 mm

#### Data collection

Agilent Xcalibur Eos diffractometer
Absorption correction: multi-scan (*CrysAlis PRO*; Agilent, 2012 ▶) *T*_min_ = 0.982, *T*_max_ = 1.000
9591 measured reflections
4431 independent reflections
3050 reflections with *I* > 2σ(*I*)
*R*_int_ = 0.038

#### Refinement

*R*[*F* ^2^ > 2σ(*F* ^2^)] = 0.055
*wR*(*F* ^2^) = 0.124
*S* = 1.03
4431 reflections
339 parameters
2 restraints
H atoms treated by a mixture of independent and constrained refinement
Δρ_max_ = 0.22 e Å^−3^
Δρ_min_ = −0.26 e Å^−3^

### 

Data collection: *CrysAlis PRO* (Agilent, 2012 ▶); cell refinement: *CrysAlis PRO*; data reduction: *CrysAlis PRO*; program(s) used to solve structure: *SHELXS97* (Sheldrick, 2008 ▶); program(s) used to refine structure: *SHELXL97* (Sheldrick, 2008 ▶); molecular graphics: *ORTEP-3* (Farrugia, 1997 ▶); software used to prepare material for publication: *SHELXL97*.

## Supplementary Material

Crystal structure: contains datablock(s) global, I. DOI: 10.1107/S1600536812031029/nk2169sup1.cif

Structure factors: contains datablock(s) I. DOI: 10.1107/S1600536812031029/nk2169Isup2.hkl

Supplementary material file. DOI: 10.1107/S1600536812031029/nk2169Isup3.cml

Additional supplementary materials:  crystallographic information; 3D view; checkCIF report

## Figures and Tables

**Table 1:** Hydrogen-bond geometry (Å, °)

*D*—H⋯*A*	*D*—H	H⋯*A*	*D*⋯*A*	*D*—H⋯*A*
O1—H1⋯O4^i^	0.84 (3)	1.91 (3)	2.750 (2)	175 (2)
O2—H2⋯O6^ii^	0.86 (3)	1.83 (3)	2.650 (2)	158 (2)
O3—H3⋯N2	0.88 (3)	1.90 (3)	2.730 (2)	157 (3)
O5—H5⋯N3^i^	0.99 (3)	1.64 (3)	2.623 (2)	174 (2)
O6—H6*A*⋯N6^iii^	0.82	1.94	2.755 (2)	173

Symmetry codes: (i) 
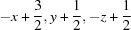
; (ii) 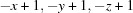; (iii) 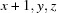.

**Fig. 1.**
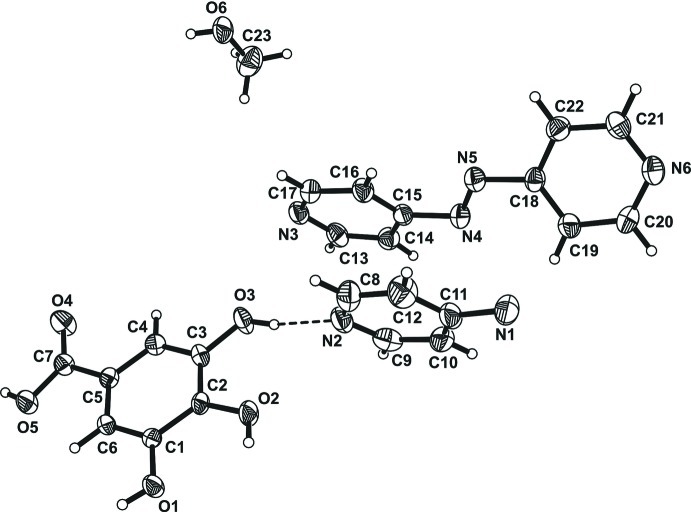
The structure of the asymmetric unit for title compound with displacement ellipsoids shown at the 50% probability level.

**Fig. 2.**
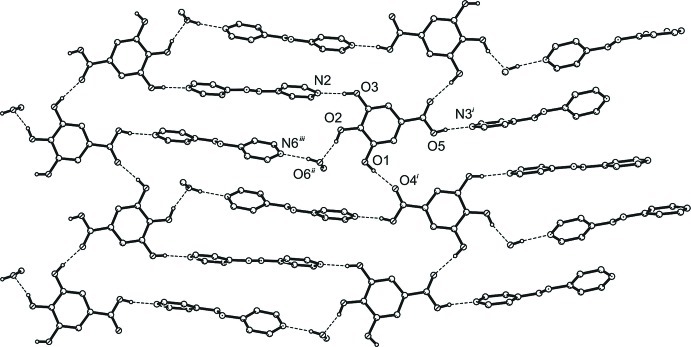
View of two-dimensional supramolecular layer. Only H atoms involved in hydrogen bonding are shown. Hydrogen bonds are shown with dashed lines. Symmetry codes: (i) 3/2 - x,1/2 + y,1/2 - z, (ii) 1 - x,1 - y,1 - z, (iii) -x,1 - y,1 - z.

## supplementary crystallographic information

### Comment

As a part of our research interest in the photosensitive compounds, we report analysis of the crystal structure of the title compound that comprises 1:1.5:1 gallic acid, 4,4'-azodipyridine and methanol molecules. The asymmetric unit of the title compound along with the atom numbering scheme is depicted in Figure 1. Gallic acid has a great potential for structure extension by the three hydroxyl groups and carboxyl function (Fazary *et al.*, 2009). 4,4'-Azodipyridine has been widely used for its bidentate type ligand ability (Zhuang *et al.*,2006; Kanoo, *et al.*, 2012) and photoswitchable properties (Qiu, *et al.*, 2011). In the crystal all components of the structure are interacting *via* an extended system of O-H_ _ _O and O-H_ _ _N hydrogen bonds, the formation of which is completely realised. The geometry of the hydrogen bonds is listed in the Table 1. The crystal structure essentially results from the packing of two-dimensional supramolecular layers in parallel orientation to the [101] plane (Figure 2). In addition, each layer is consolidated by π-π stacking interaction, which is evidenced by centroid-to-centroid distance of 3.637 Å between adjacent centrosymmetrically related AzPy rings denoted by C18 C19 C20 C21 C22 and N6 atoms.

### Experimental

AzPy were synthesized in our laboratory (Launay *et al.*, 1991). Gallic acid (0.085 g, 0.5 mmol) was dissolved in 5.0 ml MeOH and the solution was poured slowly into a methanol solution (5.0 ml) of the synthesized azodipyridine (0.092 g, 0.5 mmol). The resulting mixture was stirred for 30 min and was allowed to stand at room temperature. Slow evaporation for several weeks afforded red block-like crystals.

### Refinement

C-bound H-atoms were positioned geometrically and refined using a riding model approximation with C—H = 0.93 Å and *U*_iso_(H) = 1.2 times *U*_eq_(C). The hydroxy H-atoms were located in a difference Fourier map and refined freely. The N1 atom of the centrosymmetric AzPy presented large thermal ellipsoids, so that disordered models, in the combination with the available tools (PART and SADI) of *SHELXL97* were applied in order to better fit the electron density. It was found to be disorderd over two sites in the 0.833:0.167 (8) ratio.

### Crystal data

**Table:**

3C_10_H_8_N_4_·2C_7_H_6_O_5_·2CH_4_O	*F*(000) = 1000
*M**_r_* = 956.93	*D*_x_ = 1.411 Mg m^−^^3^
Monoclinic, *P*2_1_/*n*	Mo *K*α radiation, λ = 0.7107 Å
*a* = 13.555 (5) Å	Cell parameters from 2093 reflections
*b* = 11.711 (5) Å	θ = 2.7–29.4°
*c* = 14.213 (5) Å	µ = 0.11 mm^−^^1^
β = 93.427 (5)°	*T* = 200 K
*V* = 2252.2 (15) Å^3^	Plate, light red
*Z* = 2	0.2 × 0.2 × 0.1 mm

### Data collection

**Table:**

Agilent Xcalibur Eos diffractometer	4431 independent reflections
Radiation source: Enhance (Mo) X-ray Source	3050 reflections with *I* > 2σ(*I*)
Graphite monochromator	*R*_int_ = 0.038
Detector resolution: 16.1593 pixels mm^-1^	θ_max_ = 26.0°, θ_min_ = 2.7°
ω scans	*h* = −16→15
Absorption correction: multi-scan (*CrysAlis PRO*; Agilent, 2012)	*k* = −14→14
*T*_min_ = 0.982, *T*_max_ = 1.000	*l* = −17→12
9591 measured reflections	

### Refinement

**Table:**

Refinement on *F*^2^	Primary atom site location: structure-invariant direct methods
Least-squares matrix: full	Secondary atom site location: difference Fourier map
*R*[*F*^2^ > 2σ(*F*^2^)] = 0.055	Hydrogen site location: inferred from neighbouring sites
*wR*(*F*^2^) = 0.124	H atoms treated by a mixture of independent and constrained refinement
*S* = 1.03	*w* = 1/[σ^2^(*F*_o_^2^) + (0.0513*P*)^2^] where *P* = (*F*_o_^2^ + 2*F*_c_^2^)/3
4431 reflections	(Δ/σ)_max_ < 0.001
339 parameters	Δρ_max_ = 0.22 e Å^−^^3^
2 restraints	Δρ_min_ = −0.26 e Å^−^^3^

### Special details

**Table:**

Geometry. All e.s.d.'s (except the e.s.d. in the dihedral angle between two l.s. planes) are estimated using the full covariance matrix. The cell e.s.d.'s are taken into account individually in the estimation of e.s.d.'s in distances, angles and torsion angles; correlations between e.s.d.'s in cell parameters are only used when they are defined by crystal symmetry. An approximate (isotropic) treatment of cell e.s.d.'s is used for estimating e.s.d.'s involving l.s. planes.
Refinement. Refinement of *F*^2^ against ALL reflections. The weighted *R*-factor *wR* and goodness of fit *S* are based on *F*^2^, conventional *R*-factors *R* are based on *F*, with *F* set to zero for negative *F*^2^. The threshold expression of *F*^2^ > σ(*F*^2^) is used only for calculating *R*-factors(gt) *etc*. and is not relevant to the choice of reflections for refinement. *R*-factors based on *F*^2^ are statistically about twice as large as those based on *F*, and *R*- factors based on ALL data will be even larger.

### Fractional atomic coordinates and isotropic or equivalent isotropic displacement parameters (Å^2^)

**Table:**

	*x*	*y*	*z*	*U*_iso_*/*U*_eq_	Occ. (<1)
O1	0.52177 (11)	0.94158 (12)	0.30966 (11)	0.0315 (4)	
H1	0.5624 (19)	0.993 (2)	0.2971 (18)	0.062 (9)*	
O2	0.41107 (10)	0.75077 (13)	0.34448 (11)	0.0324 (4)	
H2	0.390 (2)	0.819 (2)	0.3522 (18)	0.061 (9)*	
O3	0.49212 (11)	0.53785 (11)	0.33081 (12)	0.0367 (4)	
H3	0.433 (2)	0.554 (2)	0.349 (2)	0.089 (11)*	
O4	0.83875 (11)	0.59942 (12)	0.23623 (12)	0.0440 (5)	
O5	0.84265 (11)	0.78510 (11)	0.20145 (11)	0.0349 (4)	
H5	0.909 (2)	0.769 (2)	0.1798 (19)	0.067 (8)*	
O6	0.68514 (11)	0.06508 (12)	0.60317 (11)	0.0395 (4)	
H6A	0.7410	0.0866	0.5943	0.065 (9)*	
N1	0.03690 (16)	0.5003 (2)	0.52750 (17)	0.0405 (10)	0.833 (8)
N1X	0.0184 (6)	0.4849 (11)	0.4631 (5)	0.039 (5)*	0.167 (8)
N2	0.31064 (13)	0.52709 (15)	0.40387 (15)	0.0383 (5)	
N3	0.47633 (13)	0.25366 (14)	0.34904 (14)	0.0329 (5)	
N4	0.18017 (12)	0.21047 (14)	0.41139 (13)	0.0325 (5)	
N5	0.16762 (13)	0.18525 (15)	0.49493 (14)	0.0341 (5)	
N6	−0.12425 (13)	0.12055 (16)	0.56342 (14)	0.0378 (5)	
C1	0.56417 (14)	0.83667 (15)	0.29803 (14)	0.0229 (5)	
C2	0.50510 (14)	0.74231 (16)	0.31668 (14)	0.0236 (5)	
C3	0.54455 (14)	0.63231 (16)	0.30990 (14)	0.0256 (5)	
C4	0.63951 (14)	0.61786 (17)	0.28240 (14)	0.0266 (5)	
H4	0.6651	0.5446	0.2771	0.032*	
C5	0.69760 (14)	0.71213 (16)	0.26244 (14)	0.0237 (5)	
C6	0.65943 (14)	0.82173 (16)	0.27075 (14)	0.0238 (5)	
H6	0.6980	0.8849	0.2580	0.029*	
C7	0.79932 (15)	0.69343 (17)	0.23271 (15)	0.0273 (5)	
C8	0.29820 (17)	0.5386 (2)	0.49523 (19)	0.0443 (6)	
H8	0.3534	0.5549	0.5350	0.053*	
C9	0.23096 (18)	0.50213 (18)	0.34829 (18)	0.0411 (6)	
H9	0.2388	0.4915	0.2843	0.049*	
C10	0.13696 (17)	0.49117 (18)	0.38069 (18)	0.0423 (6)	
H10	0.0829	0.4743	0.3396	0.051*	
C11	0.12626 (16)	0.50610 (18)	0.47611 (19)	0.0399 (6)	
C12	0.20885 (19)	0.5279 (2)	0.53435 (19)	0.0484 (7)	
H12	0.2040	0.5353	0.5991	0.058*	
C13	0.40522 (17)	0.22890 (17)	0.28328 (17)	0.0355 (6)	
H13	0.4229	0.2178	0.2217	0.043*	
C14	0.30687 (16)	0.21897 (17)	0.30187 (16)	0.0331 (5)	
H14	0.2587	0.2060	0.2536	0.040*	
C15	0.28212 (15)	0.22895 (16)	0.39459 (16)	0.0282 (5)	
C16	0.35448 (16)	0.25523 (17)	0.46419 (16)	0.0324 (5)	
H16	0.3391	0.2640	0.5267	0.039*	
C17	0.45017 (16)	0.26794 (17)	0.43762 (17)	0.0339 (5)	
H17	0.4990	0.2874	0.4836	0.041*	
C18	0.06578 (15)	0.16613 (17)	0.51343 (15)	0.0289 (5)	
C19	−0.01382 (16)	0.20737 (18)	0.45826 (17)	0.0345 (5)	
H19	−0.0049	0.2499	0.4042	0.041*	
C20	−0.10726 (16)	0.18273 (19)	0.48684 (16)	0.0367 (6)	
H20	−0.1614	0.2109	0.4509	0.044*	
C21	−0.04618 (17)	0.08113 (19)	0.61452 (16)	0.0376 (6)	
H21	−0.0569	0.0363	0.6669	0.045*	
C22	0.05013 (16)	0.10366 (18)	0.59334 (15)	0.0347 (5)	
H22	0.1030	0.0773	0.6321	0.042*	
C23	0.64365 (18)	0.0384 (2)	0.5119 (2)	0.0531 (7)	
H23A	0.6324	0.1076	0.4767	0.080*	
H23B	0.5820	−0.0008	0.5172	0.080*	
H23C	0.6884	−0.0096	0.4801	0.080*	

### Atomic displacement parameters (Å^2^)

**Table:**

	*U*^11^	*U*^22^	*U*^33^	*U*^12^	*U*^13^	*U*^23^
O1	0.0246 (8)	0.0227 (8)	0.0484 (11)	0.0012 (6)	0.0116 (7)	0.0021 (7)
O2	0.0200 (8)	0.0320 (9)	0.0462 (11)	−0.0011 (6)	0.0106 (7)	0.0002 (8)
O3	0.0309 (9)	0.0264 (8)	0.0544 (11)	−0.0063 (7)	0.0152 (8)	−0.0023 (7)
O4	0.0367 (9)	0.0286 (8)	0.0691 (13)	0.0075 (7)	0.0231 (9)	0.0008 (8)
O5	0.0245 (8)	0.0313 (8)	0.0505 (11)	0.0008 (7)	0.0163 (8)	0.0045 (7)
O6	0.0238 (9)	0.0459 (9)	0.0497 (11)	−0.0007 (7)	0.0099 (8)	0.0033 (8)
N1	0.0297 (16)	0.0532 (16)	0.0384 (19)	−0.0021 (12)	0.0018 (11)	0.0007 (13)
N2	0.0312 (11)	0.0305 (10)	0.0549 (14)	−0.0016 (8)	0.0157 (11)	0.0038 (10)
N3	0.0270 (10)	0.0294 (10)	0.0432 (12)	−0.0012 (8)	0.0098 (9)	0.0027 (9)
N4	0.0252 (10)	0.0367 (10)	0.0360 (12)	−0.0015 (8)	0.0056 (9)	−0.0010 (9)
N5	0.0282 (10)	0.0387 (10)	0.0360 (12)	−0.0020 (8)	0.0056 (9)	0.0026 (9)
N6	0.0315 (11)	0.0436 (11)	0.0392 (12)	−0.0022 (9)	0.0102 (10)	−0.0029 (10)
C1	0.0211 (10)	0.0229 (10)	0.0247 (12)	0.0007 (8)	0.0020 (9)	0.0013 (9)
C2	0.0175 (10)	0.0303 (11)	0.0233 (11)	−0.0013 (8)	0.0049 (9)	−0.0006 (9)
C3	0.0248 (11)	0.0259 (11)	0.0264 (12)	−0.0071 (9)	0.0043 (9)	−0.0008 (9)
C4	0.0260 (11)	0.0251 (11)	0.0290 (12)	0.0017 (9)	0.0037 (10)	−0.0036 (9)
C5	0.0214 (10)	0.0277 (10)	0.0224 (11)	0.0004 (9)	0.0040 (9)	−0.0009 (9)
C6	0.0214 (11)	0.0246 (10)	0.0256 (12)	−0.0028 (8)	0.0042 (9)	0.0027 (9)
C7	0.0262 (11)	0.0285 (11)	0.0277 (12)	0.0007 (9)	0.0068 (10)	−0.0018 (10)
C8	0.0334 (14)	0.0476 (15)	0.0518 (18)	−0.0066 (11)	0.0013 (13)	−0.0032 (13)
C9	0.0535 (16)	0.0340 (13)	0.0365 (15)	0.0042 (11)	0.0079 (13)	0.0055 (11)
C10	0.0348 (14)	0.0366 (13)	0.0541 (18)	−0.0029 (10)	−0.0087 (13)	0.0043 (12)
C11	0.0286 (13)	0.0331 (12)	0.0596 (18)	0.0022 (10)	0.0152 (13)	0.0077 (12)
C12	0.0465 (16)	0.0569 (16)	0.0432 (16)	0.0024 (12)	0.0133 (13)	−0.0028 (13)
C13	0.0370 (13)	0.0336 (12)	0.0372 (14)	−0.0034 (10)	0.0139 (11)	−0.0009 (11)
C14	0.0325 (13)	0.0341 (12)	0.0327 (14)	−0.0016 (10)	0.0015 (11)	−0.0001 (10)
C15	0.0244 (11)	0.0245 (10)	0.0360 (13)	−0.0001 (9)	0.0049 (10)	0.0016 (10)
C16	0.0292 (12)	0.0375 (12)	0.0313 (13)	−0.0018 (10)	0.0076 (10)	0.0010 (10)
C17	0.0266 (12)	0.0371 (12)	0.0379 (14)	−0.0028 (9)	0.0014 (11)	0.0026 (11)
C18	0.0237 (11)	0.0295 (11)	0.0339 (13)	0.0010 (9)	0.0051 (10)	−0.0044 (10)
C19	0.0315 (13)	0.0365 (12)	0.0361 (14)	0.0006 (10)	0.0076 (11)	0.0026 (11)
C20	0.0257 (12)	0.0434 (13)	0.0410 (15)	0.0040 (10)	0.0017 (11)	−0.0029 (12)
C21	0.0387 (14)	0.0421 (13)	0.0330 (14)	−0.0031 (11)	0.0107 (11)	0.0010 (11)
C22	0.0319 (12)	0.0402 (13)	0.0318 (13)	0.0020 (10)	0.0011 (10)	0.0012 (11)
C23	0.0410 (15)	0.0501 (15)	0.068 (2)	0.0060 (12)	−0.0027 (14)	−0.0099 (14)

### Geometric parameters (Å, º)

**Table:**

O1—H1	0.84 (3)	C5—C6	1.392 (3)
O1—C1	1.371 (2)	C5—C7	1.482 (3)
O2—H2	0.86 (3)	C6—H6	0.9300
O2—C2	1.360 (2)	C8—H8	0.9300
O3—H3	0.88 (3)	C8—C12	1.368 (3)
O3—C3	1.357 (2)	C9—H9	0.9300
O4—C7	1.224 (2)	C9—C10	1.386 (3)
O5—H5	0.99 (3)	C10—H10	0.9300
O5—C7	1.314 (2)	C10—C11	1.384 (3)
O6—H6A	0.8154	C11—C12	1.376 (3)
O6—C23	1.417 (3)	C12—H12	0.9300
N1—N1^i^	1.232 (4)	C13—H13	0.9300
N1—C11	1.453 (3)	C13—C14	1.379 (3)
N1X—N1X^i^	1.239 (10)	C14—H14	0.9300
N1X—C11	1.483 (9)	C14—C15	1.384 (3)
N2—C8	1.326 (3)	C15—C16	1.385 (3)
N2—C9	1.332 (3)	C16—H16	0.9300
N3—C13	1.334 (3)	C16—C17	1.380 (3)
N3—C17	1.339 (3)	C17—H17	0.9300
N4—N5	1.245 (2)	C18—C19	1.383 (3)
N4—C15	1.433 (3)	C18—C22	1.378 (3)
N5—C18	1.438 (3)	C19—H19	0.9300
N6—C20	1.341 (3)	C19—C20	1.383 (3)
N6—C21	1.330 (3)	C20—H20	0.9300
C1—C2	1.399 (3)	C21—H21	0.9300
C1—C6	1.381 (3)	C21—C22	1.383 (3)
C2—C3	1.400 (3)	C22—H22	0.9300
C3—C4	1.378 (3)	C23—H23A	0.9600
C4—H4	0.9300	C23—H23B	0.9600
C4—C5	1.395 (3)	C23—H23C	0.9600
			
C1—O1—H1	109.4 (17)	C10—C11—N1X	91.0 (3)
C2—O2—H2	115.5 (17)	C12—C11—N1	112.3 (2)
C3—O3—H3	112.6 (19)	C12—C11—N1X	150.0 (3)
C7—O5—H5	112.8 (14)	C12—C11—C10	119.0 (2)
C23—O6—H6A	104.3	C8—C12—C11	118.7 (2)
N1^i^—N1—C11	110.5 (3)	C8—C12—H12	120.6
N1X^i^—N1X—C11	106.9 (10)	C11—C12—H12	120.6
C8—N2—C9	117.2 (2)	N3—C13—H13	118.3
C13—N3—C17	117.73 (19)	N3—C13—C14	123.4 (2)
N5—N4—C15	112.51 (19)	C14—C13—H13	118.3
N4—N5—C18	113.50 (19)	C13—C14—H14	121.1
C21—N6—C20	117.52 (19)	C13—C14—C15	117.9 (2)
O1—C1—C2	115.87 (17)	C15—C14—H14	121.1
O1—C1—C6	123.58 (16)	C14—C15—N4	115.9 (2)
C6—C1—C2	120.54 (17)	C14—C15—C16	119.80 (19)
O2—C2—C1	123.65 (17)	C16—C15—N4	124.3 (2)
O2—C2—C3	117.08 (16)	C15—C16—H16	121.1
C1—C2—C3	119.23 (17)	C17—C16—C15	117.7 (2)
O3—C3—C2	121.84 (17)	C17—C16—H16	121.1
O3—C3—C4	118.18 (17)	N3—C17—C16	123.4 (2)
C4—C3—C2	119.97 (17)	N3—C17—H17	118.3
C3—C4—H4	119.7	C16—C17—H17	118.3
C3—C4—C5	120.58 (18)	C19—C18—N5	124.54 (19)
C5—C4—H4	119.7	C22—C18—N5	115.48 (19)
C4—C5—C7	119.14 (17)	C22—C18—C19	119.97 (19)
C6—C5—C4	119.70 (18)	C18—C19—H19	121.4
C6—C5—C7	121.15 (17)	C18—C19—C20	117.2 (2)
C1—C6—C5	119.95 (17)	C20—C19—H19	121.4
C1—C6—H6	120.0	N6—C20—C19	123.8 (2)
C5—C6—H6	120.0	N6—C20—H20	118.1
O4—C7—O5	123.09 (19)	C19—C20—H20	118.1
O4—C7—C5	122.20 (18)	N6—C21—H21	118.5
O5—C7—C5	114.71 (17)	N6—C21—C22	123.1 (2)
N2—C8—H8	118.1	C22—C21—H21	118.5
N2—C8—C12	123.8 (2)	C18—C22—C21	118.3 (2)
C12—C8—H8	118.1	C18—C22—H22	120.8
N2—C9—H9	118.2	C21—C22—H22	120.8
N2—C9—C10	123.5 (2)	O6—C23—H23A	109.5
C10—C9—H9	118.2	O6—C23—H23B	109.5
C9—C10—H10	121.1	O6—C23—H23C	109.5
C11—C10—C9	117.8 (2)	H23A—C23—H23B	109.5
C11—C10—H10	121.1	H23A—C23—H23C	109.5
N1—C11—N1X	37.8 (3)	H23B—C23—H23C	109.5
C10—C11—N1	128.8 (2)		
			
O1—C1—C2—O2	0.2 (3)	C3—C4—C5—C6	0.3 (3)
O1—C1—C2—C3	−177.56 (18)	C3—C4—C5—C7	−179.79 (19)
O1—C1—C6—C5	178.69 (18)	C4—C5—C6—C1	−0.5 (3)
O2—C2—C3—O3	−0.6 (3)	C4—C5—C7—O4	−9.3 (3)
O2—C2—C3—C4	−179.80 (19)	C4—C5—C7—O5	169.88 (19)
O3—C3—C4—C5	−178.34 (19)	C6—C1—C2—O2	179.48 (19)
N1^i^—N1—C11—N1X	−12.9 (8)	C6—C1—C2—C3	1.7 (3)
N1^i^—N1—C11—C10	−12.1 (4)	C6—C5—C7—O4	170.6 (2)
N1^i^—N1—C11—C12	168.9 (3)	C6—C5—C7—O5	−10.2 (3)
N1—C11—C12—C8	−178.3 (2)	C7—C5—C6—C1	179.6 (2)
N1X^i^—N1X—C11—N1	12.6 (8)	C8—N2—C9—C10	1.9 (3)
N1X^i^—N1X—C11—C10	−166.8 (14)	C9—N2—C8—C12	−0.9 (3)
N1X^i^—N1X—C11—C12	16 (2)	C9—C10—C11—N1	179.4 (2)
N1X—C11—C12—C8	179.4 (10)	C9—C10—C11—N1X	179.9 (5)
N2—C8—C12—C11	−1.3 (4)	C9—C10—C11—C12	−1.7 (3)
N2—C9—C10—C11	−0.6 (3)	C10—C11—C12—C8	2.6 (3)
N3—C13—C14—C15	−3.8 (3)	C13—N3—C17—C16	1.8 (3)
N4—N5—C18—C19	−21.4 (3)	C13—C14—C15—N4	−175.97 (17)
N4—N5—C18—C22	159.80 (19)	C13—C14—C15—C16	4.0 (3)
N4—C15—C16—C17	178.45 (18)	C14—C15—C16—C17	−1.5 (3)
N5—N4—C15—C14	158.93 (18)	C15—N4—N5—C18	−179.91 (16)
N5—N4—C15—C16	−21.0 (3)	C15—C16—C17—N3	−1.5 (3)
N5—C18—C19—C20	−179.0 (2)	C17—N3—C13—C14	0.9 (3)
N5—C18—C22—C21	−179.25 (19)	C18—C19—C20—N6	−0.9 (3)
N6—C21—C22—C18	−2.6 (3)	C19—C18—C22—C21	1.9 (3)
C1—C2—C3—O3	177.32 (19)	C20—N6—C21—C22	1.6 (3)
C1—C2—C3—C4	−1.9 (3)	C21—N6—C20—C19	0.3 (3)
C2—C1—C6—C5	−0.6 (3)	C22—C18—C19—C20	−0.2 (3)
C2—C3—C4—C5	0.9 (3)		

Symmetry code: (i) −*x*, −*y*+1, −*z*+1.

### Hydrogen-bond geometry (Å, º)

**Table:**

*D*—H···*A*	*D*—H	H···*A*	*D*···*A*	*D*—H···*A*
O1—H1···O4^ii^	0.84 (3)	1.91 (3)	2.750 (2)	175 (2)
O2—H2···O6^iii^	0.86 (3)	1.83 (3)	2.650 (2)	158 (2)
O3—H3···N2	0.88 (3)	1.90 (3)	2.730 (2)	157 (3)
O5—H5···N3^ii^	0.99 (3)	1.64 (3)	2.623 (2)	174 (2)
O6—H6*A*···N6^iv^	0.82	1.94	2.755 (2)	173

Symmetry codes: (ii) −*x*+3/2, *y*+1/2, −*z*+1/2; (iii) −*x*+1, −*y*+1, −*z*+1; (iv) *x*+1, *y*, *z*.
